# Glycoproteomic Signatures of IgG Subclass *N*‐Glycosylation Reveal Molecular Endotypes in Idiopathic Inflammatory Myopathies

**DOI:** 10.1002/mco2.70987

**Published:** 2026-09-22

**Authors:** Tong Wu, Yanhong Li, Yingying Ling, Yinlan Wu, Jing Zhao, Lu Cheng, Chunyu Tan, Yi Zhang, Yong Zhang, Yi Liu

**Affiliations:** ^1^ Department of Rheumatology and Immunology Laboratory of Rheumatology and Immunology West China Hospital Sichuan University Chengdu China; ^2^ Institute of Immunology and Inflammation Frontiers Science Center For Disease‐related Molecular Network West China Hospital Sichuan University Chengdu China; ^3^ Department of Pulmonary and Critical Care Medicine State Key Laboratory of Respiratory Health and Multimorbidity West China Hospital Sichuan University Chengdu China; ^4^ Division of Pancreatic Surgery Department of General Surgery West China Hospital Sichuan University Chengdu China; ^5^ West China Lecheng Hospital Sichuan University Boao Hainan China

**Keywords:** idiopathic inflammatory myopathies, immunoglobulin G, *N*‐glycosylation, glycoproteomics, liquid chromatography‐tandem mass spectrometry

## Abstract

Idiopathic inflammatory myopathies (IIMs) are heterogeneous autoimmune diseases for which robust molecular classifiers are lacking. In this retrospective cross‐sectional study, we profiled immunoglobulin G (IgG) subclass‐specific *N*‐glycosylation in IIMs and investigated their associations with clinical phenotypes and histopathological features. Using liquid chromatography‐tandem mass spectrometry (LC‐MS/MS) with an electron‐transfer/higher energy collisional dissociation‐stepped collision energy higher energy collisional dissociation (EThcD‐sceHCD) fragmentation strategy, we analyzed plasma samples from 145 patients with IIMs and 52 age‐ and sex‐comparable healthy controls (HCs). Compared with HCs, patients with IIMs showed increased core fucosylation and significant alterations in 13 site‐specific IgG glycopeptides. Unsupervised clustering identified three glycosylation‐defined endotypes with muscle‐, cutaneous‐, and joint‐dominant phenotypes, each characterized by distinct glycoform patterns, clinical manifestations, and serological profiles. Specific IgG2 glycoforms were associated with markers of muscle injury and membrane attack complex (C5b‐9) deposition, suggesting a link between IgG glycosylation and complement‐mediated tissue damage. A seven‐glycopeptide multinomial logistic regression model and endotype‐specific nomograms were developed for exploratory cluster assignment, with moderate discrimination in the internal validation cohort (macro‐averaged area under the curve [macro‐AUC], 0.74). These findings identify IgG subclass‐specific *N*‐glycosylation as a molecular layer underlying IIM heterogeneity beyond conventional clinical classification and provide a glycoproteomic framework for biomarker‐driven stratification, histopathological interpretation, and future mechanistic investigation.

## Graphical abstract

• EThcD‐sceHCD‐MS/MS and updated PANDA software decode IgG N‐glycosylation in IIMs.

• Unsupervised clustering reveals three novel glyco‐endotypes matching clinical phenotypes.

• A seven‐glycopeptide model enables robust endotype prediction beyond conventional subtypes.

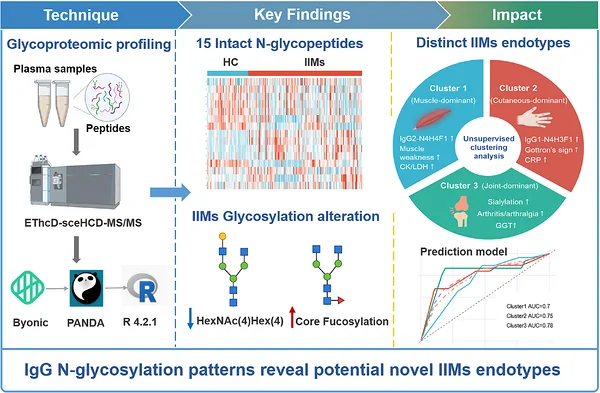

## 1 Introduction

Idiopathic inflammatory myopathies (IIMs) are a diverse group of autoimmune disorders characterized by chronic muscle inflammation and variable extramuscular involvement, including cutaneous, pulmonary, articular, gastrointestinal, and cardiovascular manifestations [1]. This clinical heterogeneity poses substantial diagnostic and therapeutic challenges because patients assigned the same diagnostic labels may exhibit distinct clinical manifestations, treatment response, and prognoses. Although the recognition of myositis‐specific autoantibodies (MSAs) has enhanced the clinical stratification of IIMs, current classification systems, such as the European League Against Rheumatism/American College of Rheumatology (EULAR/ACR) classification criteria, do not fully capture the biological heterogeneity of IIMs, with up to 30% of patients remaining unclassified [2, 3]. These limitations highlight the need for molecular stratification approaches that can complement existing clinical and autoantibody‐based classifications and more accurately reflect disease‐relevant immune pathways.

Post‐translational modifications of immunoglobulin G (IgG), particularly the *N*‐glycosylation of the fragment crystallizable (Fc) region, have emerged as important regulators of humoral immune function [4, 5]. Fc N‐glycans modulate the interactions of IgG with Fcγ receptors (FcγRs) and complement components, thereby influencing antibody‐dependent cellular cytotoxicity (ADCC) and complement‐mediated inflammation [6, 7, 8]. Specific Fc glycan features, including core fucosylation, galactosylation, and sialylation, can shift IgG effector functions toward pro‐inflammatory or anti‐inflammatory states [9]. Thus, IgG *N*‐glycosylation may serve not only as a biomarker of immune dysregulation but also as a molecular link between circulating autoantibodies and tissue‐level inflammatory injury.

Accumulating evidence from autoimmune diseases supports the clinical relevance of IgG glycosylation. In rheumatoid arthritis (RA), specific IgG glycoforms have been associated with disease activity and may predict the response to anti‐TNF therapy [10, 11, 12]. Similarly, in systemic lupus erythematosus (SLE), galactose‐deficient IgG glycoforms have been implicated in immune‐complex deposition and tissue inflammation [13]. These findings suggest that IgG glycosylation is not merely a secondary consequence of inflammation but may constitute a biologically informative signature linking humoral immunity to tissue injury. However, glycoproteomic studies in IIMs remain limited, particularly regarding whether IgG subclass‐specific *N*‐glycosylation captures the broader molecular heterogeneity of IIMs [14].

In this study, we profiled plasma IgG subclass‐specific intact IGPs in patients with IIMs and matched healthy controls using an intact glycopeptide (IGPs)‐based glycoproteomic strategy. We aimed to characterize disease‐associated alterations in IgG glycosylation, identify glycosylation‐defined molecular endotypes through unsupervised clustering, and evaluate their associations with clinical phenotypes, serological profiles, disease activity, and histopathological complement deposition. By integrating glycoproteomic, clinical, and histopathological data, we propose a glyco‐phenotyping framework for understanding heterogeneity in IIMs and provide a foundation for biomarker‐based stratification and future mechanistic studies.

## 2 Results

### 2.1 Patients With IIMs Exhibit Distinct IgG *N*‐Glycosylation Profiles Compared With Healthy Controls

This study included 145 patients with IIMs (IIMs group) and 52 age‐ and sex‐comparable healthy controls (HC group) (Table 1). Glycoproteomic analysis identified 11 structurally distinct IgG *N*‐glycans, nine of which differed significantly between the IIMs and HC groups (Table S1). These structures were labeled based on their composition: hexose (H), *N*‐acetylhexosamine (N), fucose (F), and *N*‐acetylneuraminic acid (A).

**TABLE 1:** Baseline characteristics of the study population.

Variables	HC (*n* = 52)	IIMs (*n* = 145)	*p* value
Demographic data			
Female, *n* (%)	34 (65.38)	114 (78.62)	0.0640
Age, years (mean, SD)	50.31 (8.59)	48.53 (11.66)	0.3163
BMI, kg/m^2^ (mean, SD)	22.20 (2.49)	22.44 (3.26)	0.5864
Comorbidities, *n* (%)			
Hypertension	—	12 (8.28)	—
Diabetes	—	11 (7.59)	—
Cardiovascular diseases	—	4 (2.76)	—
Diagnosis, *n* (%)			
ADM	—	24 (16.55)	—
ASS	—	35 (24.13)	—
DM	—	62 (42.76)	—
IMNM	—	24 (16.55)	—

Abbreviations: ADM, amyopathic dermatomyositis; ASS, anti‐synthetase syndrome; BMI, body mass index; DM, dermatomyositis; HC, healthy control; IIMs, idiopathic inflammatory myopathies; IMNM, immune‐mediated necrotizing myopathy.

**FIGURE 1:**
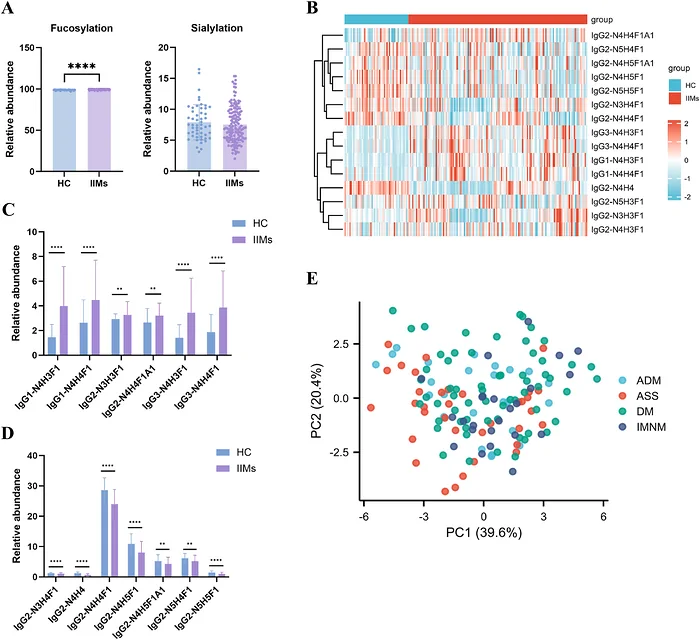
Distinct IgG *N*‐glycosylation profiles in healthy controls and patients with IIMs. (A) Comparison of total core fucosylation and sialylation between healthy controls (HCs) and patients with idiopathic inflammatory myopathies (IIMs). (B) Heatmap of 15 intact IgG N‐glycopeptide features in the two groups. (C) Six intact IgG N‐glycopeptides that were upregulated in patients with IIMs. (D) Seven intact IgG N‐glycopeptides that were downregulated in patients with IIMs. (E) PCA plot indicates limited discriminatory power among conventional IIMs subtypes. Asterisks represent the *p*‐value as follows: *p < 0.05, **p < 0.01, ***p < 0.001, ****p < 0.0001.

The IIMs group had significantly higher levels of core fucosylation (99.34% ± 0.45% vs. 98.74% ± 0.38%, *p* < 0.0001) (Figure 1A) and lower abundance of the N4H4 glycan structure (0.66% ± 0.45% vs. 1.26% ± 0.38%, *p* < 0.0001) (Table S1). Although total sialylation showed no intergroup difference (7.54% ± 2.94% vs. 7.91% ± 2.85%, *p* = 0.3628) (Figure 1A), specific sialylated *N*‐glycans differed markedly, particularly in N4H4F1A1 (3.21% ± 1.01% vs. 2.66% ± 1.13%, *p* = 0.0013) and N4H5F1A1 (4.33% ± 2.19% vs. 5.26% ± 2.11%, *p* = 0.0022) (Table S1). Site‐specific analysis identified 15 quantitatively detectable IGPs with distinct abundance patterns (Figure 1B). Compared with HCs, patients with IIMs demonstrated significantly higher abundances of six IGPs, including IgG1‐N4H3F1 (3.99% ± 3.20% vs. 1.47% ± 1.03%, *p* < 0.0001) and IgG3‐N4H4F1 (3.87% ± 2.96% vs. 1.87% ± 1.44%, *p* < 0.0001) (Figure 1C). Conversely, seven IGPs, including IgG2‐N5H4F1 (5.24% ± 1.95% vs. 6.16% ± 1.60%, *p* = 0.0028), were less abundant (Figure 1D).

Subgroup analysis among conventional IIMs subtypes revealed divergent glycosylation patterns (Table S2). Patients with amyopathic dermatomyositis (ADM) showed the highest levels of core fucosylation (ADM: 99.55% ± 0.35% vs. IMNM: 98.87% ± 0.49%, *p* < 0.0001), whereas patients with dermatomyositis (DM) exhibited increased sialylation (DM: 8.18% ± 3.03% vs. ASS: 6.43% ± 2.77%, *p* = 0.0156). Notably, IgG2 *N*‐glycans displayed particularly pronounced differences. IgG2‐N4H4 and IgG2‐N4H4F1 showed their highest abundances in IMNM (1.13% ± 0.49% and 28.27% ± 4.35%, respectively; both overall *p* < 0.0001), whereas IgG2‐N3H3F1 and IgG2‐N4H3F1 were most abundant in ASS (4.19% ± 1.08% and 30.03% ± 7.16%; overall *p* < 0.0001 and *p* = 0.0051, respectively). However, principal component analysis (PCA) demonstrated limited discrimination among conventional IIMs subtypes (Figure 1E). This limited separation highlights considerable overlap in glycosylation profiles across diagnostic categories, suggesting that current clinical classifications do not fully capture the molecular heterogeneity reflected by IgG glycosylation patterns. These findings support further evaluation of glyco‐phenotype‐based reclassification strategies.

### 2.2 Unsupervised Clustering Identifies Three Novel Subgroups of Patients With Distinct IgG *N*‐Glycosylation Profiles

**FIGURE 2:**
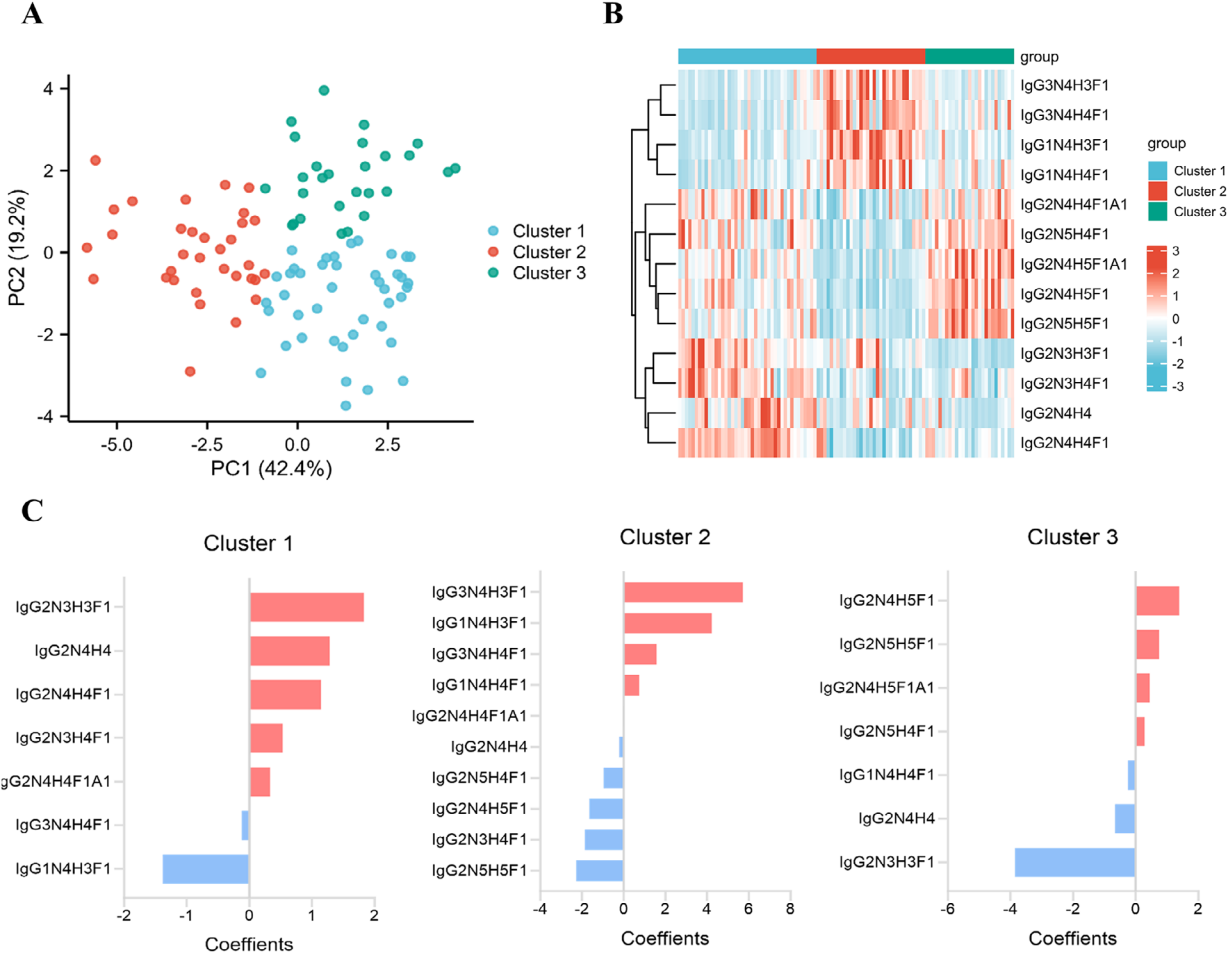
IgG *N*‐glycosylation profiles of three clusters identified by unsupervised clustering analysis. (A) PCA showing separation of the three clusters based on 13 differentially abundant intact N‐glycopeptides (IGPs). (B) Heatmap displays the differential abundance of IGPs in IgGs across the three clusters. (C) LASSO regression identifying the most and least relevant IGPs associated with each cluster. The y‐axis represents the selected glycans, color‐coded by the direction of association, and the x‐axis shows the corresponding coefficients.

To explore an *N*‐glycosylation‐based molecular classification, we performed unsupervised clustering analysis of 102 patients in the training set (70% of the cohort) using 13 differentially abundant site‐specific *N*‐glycopeptides. K‐means clustering and silhouette coefficient analysis identified three clusters, with mean coefficients of 0.181 (Cluster 1, *n* = 42), 0.256 (Cluster 2, *n* = 33), and 0.237 (Cluster 3, *n* = 27), and an overall average silhouette width of 0.220. PCA visualization showed greater separation among these clusters than among conventional subtypes (Figure 2A).

Heatmap visualization revealed distinct IgG *N*‐glycosylation patterns across the three clusters (Figure 2B). Cluster 1 (*n* = 42) was enriched in IgG2‐associated glycoforms, with particularly high levels of IgG2‐N4H4F1 (42.68% ± 4.69%) and IgG2‐N3H3F1 (6.70% ± 2.21%). In this cluster, IgG1‐ and IgG3‐associated glycoforms were significantly depleted relative to the other clusters. Least absolute shrinkage and selection operator (LASSO) regression analysis further highlighted IgG2‐N3H3F1 and IgG2‐N4H4 as the glycoforms most strongly associated with Cluster 1. Cluster 2 (*n* = 33) was characterized by high abundances of IgG1‐ and IgG3‐associated glycoforms, including IgG1‐N4H3F1 (11.07% ± 4.26%) and IgG3‐N4H3F1 (9.54% ± 4.75%), together with lower IgG2‐N5H5F1 abundance (0.71% ± 0.35%). These patterns were consistent with the LASSO‐selected features. In contrast, Cluster 3 (*n* = 27) displayed a high‐sialylation phenotype (14.23% ± 2.96%), characterized particularly by enrichment of IgG2‐N4H5F1A1 (8.91% ± 2.57%). LASSO regression confirmed IgG2‐N4H5F1 as the principal positive driver of this cluster, whereas IgG2‐N3H3F1 had the strongest negative association (Figure 2C).

**FIGURE 3:**
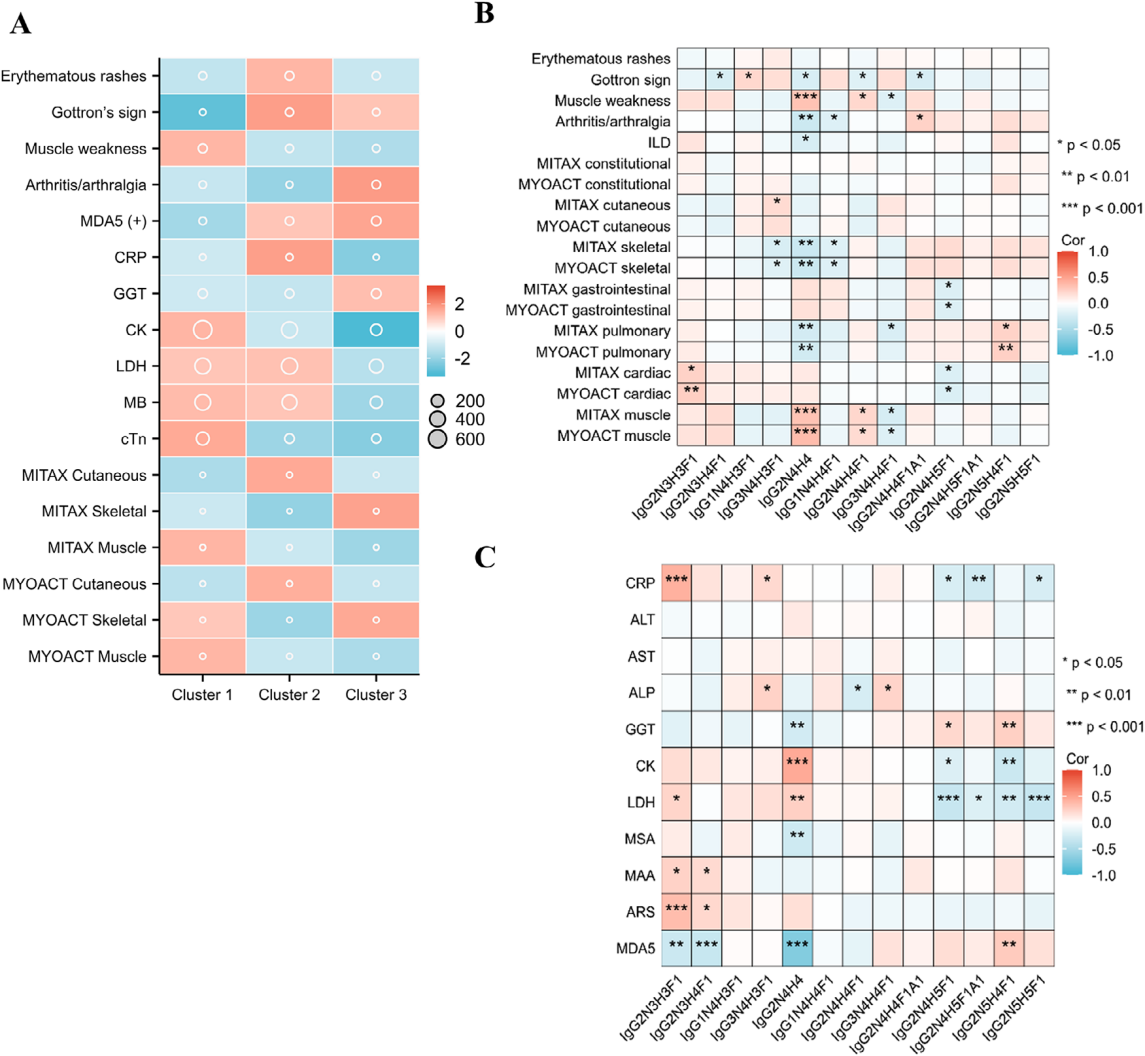
Integrated analysis of clinical phenotypes and IgG glycopeptide correlations across three clusters. (A) Heatmap showing the clinical phenotype of the three clusters: Cluster 1, muscle‐dominant phenotype; Cluster 2, cutaneous‐dominant phenotype; Cluster 3, joint‐dominant phenotype. Colors represent the difference between each cluster and the remaining patients (red, positive; blue, negative), calculated as the ratio of the cluster‐specific value to the value for all patients. When the ratio was < 1, the negative reciprocal was used and shown in blue. (B and C) Spearman correlation analyses between 13 differentially abundant intact IgG N‐glycopeptides and clinical manifestations (B) or laboratory variables (C) in patients with IIMs. Asterisks represent the *p*‐value as follows: *p < 0.05, **p < 0.01, ***p < 0.001, ****p < 0.0001.

Comprehensive clinical profiling revealed distinct phenotypic patterns among the three glycosylation‐defined clusters (Table 2). Cluster 1 exhibited muscle‐dominant characteristics, with the highest prevalence of proximal muscle weakness (54.76%, *p* = 0.0338) and the highest creatine kinase (CK) levels (220.0 [42.0–959.0] IU/L, *p* = 0.0046). These features corresponded with heightened Myositis Disease Activity Assessment Tool (MDAAT) muscle scores. Cluster 2 showed cutaneous predominance, with a higher prevalence of Gottron's sign (36.36%, *p* = 0.0064) and a higher cutaneous activity score. Cluster 3 had prominent articular involvement, with arthritis/arthralgia in 44.44% of patients (*p* = 0.0153), despite relatively preserved muscle integrity and significantly lower muscle enzyme levels. This cluster showed a consistent elevation in MDAAT skeletal scores (Figure 3A).

**TABLE 2:** Clinical characteristics of the three IIMs clusters defined by distinct IgG *N*‐glycosylation profiles.

Variables	Cluster 1 (*n* = 42)	Cluster 2 (*n* = 33)	Cluster 3 (*n* = 27)	*p* value
Demographic data				
Female, *n* (%)	32 (76.19)	26 (78.79)	21 (77.78)	> 0.9999
Age, years (mean, SD)^b^, ^c^	50.12 (11.15)	53.88 (8.82)	40.44 (11.61)	< 0.0001
BMI, kg/m^2^ (mean, SD)	23.29 (3.21)	21.74 (3.26)	22.42 (3.16)	0.1361
Disease course, months (median, IQR)	7 (2,24)	8 (2,24)	4.5 (2,6.5)	0.3275
Comorbidities, *n* (%)				
Hypertension	3 (7.14)	3 (9.09)	2 (7.41)	> 0.9999
Diabetes	4 (9.52)	2 (6.06)	2 (7.41)	0.8997
Cardiovascular disease	2 (4.76)	1 (3.03)	0	0.7821
Diagnosis, *n* (%)				
ASS^b^, ^c^	12 (28.57)	11 (33.33)	2 (7.41)	0.0425
ADM	3 (7.14)	8 (24.24)	6 (22.22)	0.0808
DM^b^	14 (33.33)	12 (36.36)	17 (62.96)	0.0457
IMNM^a^, ^b^	13 (30.95)	2 (6.06)	2 (7.41)	0.0074
General				
Fever, *n* (%)	2 (4.76)	3 (9.09)	3 (11.11)	0.6491
Cutaneous				
Erythematous rashes, *n* (%)	14 (33.33)	18 (54.55)	10 (37.04)	0.1609
Heliotrope rash, *n* (%)	2 (4.76)	6 (18.18)	1 (3.70)	0.0927
Gottron's sign, *n* (%)^a^	3 (7.14)	12 (36.36)	6 (22.22)	0.0064
V sign, *n* (%)	6 (14.29)	3 (9.09)	0	0.1168
Shawl sign, *n* (%)	1 (2.38)	3 (9.09)	1 (3.7)	0.5166
Mechanic's hands, *n* (%)	3 (7.14)	3 (9.09)	4 (14.81)	0.6380
Periungual erythema, *n* (%)	3 (7.14)	0	4 (14.81)	0.0633
Muscle weakness, *n* (%)^b^	23 (54.76)	11 (33.33)	7 (25.93)	0.0338
Myalgia, *n* (%)	12 (28.57)	6 (18.18)	5 (18.52)	0.5235
Arthritis/arthralgia, *n* (%)^c^	9 (21.43)	4 (12.12)	12 (44.44)	0.0153
Dysphagia, *n* (%)	5 (11.90)	5 (15.15)	3 (11.11)	0.8676
Pulmonary				
ILD, *n* (%)	28 (66.67)	26 (78.79)	20 (74.07)	0.5413
RP‐ILD, *n* (%)	3 (7.14)	4 (12.12)	3 (11.11)	0.7642
Cardiovascular				
Pericardial effusion, *n* (%)	8 (19.05)	6 (18.18)	2 (7.14)	0.4242
Arrhythmia, *n* (%)	6 (14.29)	2 (6.06)	1 (3.70)	0.3381
Pulmonary infection, *n* (%)	11 (26.19)	14 (42.42)	6 (22.22)	0.1970
Laboratory findings				
ANA (+), *n* (%)	25 (59.52)	21 (63.64)	12 (44.44)	0.3206
MSAs (+), *n* (%)	34 (80.95)	30 (90.91)	23 (85.19)	0.5610
MAAs (+), *n* (%)	26 (61.90)	19 (57.58)	16 (59.26)	0.9664
ARS (+), *n* (%)	12 (28.57)	12 (36.36)	4 (14.81)	0.1842
SRP (+), *n* (%)	5 (11.90)	1 (3.03)	1 (3.70)	0.3638
MDA5 (+), *n* (%)^b^	10 (23.81)	15 (45.45)	19 (70.37)	0.0006
TIF1γ (+), *n* (%)	3 (7.14)	1 (3.03)	1 (3.70)	0.8526
HMGCR (+), *n* (%)	3 (7.14)	1 (3.03)	0	0.4571
CRP, mg/L (median, IQR)^c^	4.50 (2.23, 12.73)	5.44 (2.35, 11.55)	2.63 (1.69, 4.83)	0.0150
ALT, U/L (median, IQR)	47.00 (21.75, 87.25)	27.50 (16.25, 72.25)	29.00 (21.00, 85.00)	0.3764
AST, U/L (median, IQR)	34.50 (22.25, 54.25)	31.00 (19.50, 76.50)	27.50 (18.75, 75.00)	0.7812
ALP, U/L (median, IQR)	62.00 (49.75, 81.25)	70.00 (64.00, 78.00)	74.00 (53.00, 94.00)	0.3026
GGT, U/L (median, IQR)^c^	30.00 (17.50, 73.00)	23.00 (14.00, 45.00)	49.00 (30.00, 105.00)	0.0205
CK, IU/L (median, IQR)	220.0 (42.00, 959.0)	100.0 (29.00, 508.0)	38.5 (25.50, 94.50)	0.0046
LDH, IU/L (median, IQR)	323.0 (245.0, 439.8)	314.0 (256.0, 516.0)	237.0 (194.0, 284.0)	0.0012
MB, ng/mL (median, IQR)^d^	124.6 (38.16, 518.3)	54.21 (23.71, 257.6)	29.53 (21.00, 61.33)	0.0123
CK‐MB, ng/mL (median, IQR)^e^	4.60 (1.68, 43.90)	2.09 (1.14, 16.44)	1.87 (1.13, 3.71)	0.0520
cTn, ng/L (median, IQR)^f^	53.65 (13.20, 260.3)	23.70 (9.625, 108.6)	15.80 (8.00, 40.90)	0.0340
WBC, ×10^9^/L (mean, SD)	8.78 (4.15)	7.51 (3.44)	7.97 (3.08)	0.3178
PMN, ×10^9^/L (mean, SD)	6.44 (3.61)	5.48 (3.01)	6.02 (2.83)	0.4434
LYM, ×10^9^/L (mean, SD)	1.61 (0.90)	1.34 (0.67)	1.30 (0.80)	0.2026
MITAX scores (mean, SD)	0.23 (0.12)	0.24 (0.15)	0.21 (0.14)	0.7970
Constitutional	2.45 (1.49)	2.58 (1.60)	2.56 (2.21)	0.7882
Cutaneous	0.90 (1.25)	2.33 (2.56)	1.37 (1.25)	0.0193
Skeletal	0.58 (0.99)	0.27 (0.63)	0.96 (1.09)	0.0068
Gastrointestinal	0.52 (1.61)	0.55 (1.70)	0.59 (1.87)	0.9272
Pulmonary	3.31 (3.62)	2.82 (3.70)	3.67 (3.72)	0.4722
Cardiovascular	4.02 (3.59)	4.55 (4.25)	3.22 (3.71)	0.4651
Muscle	2.48 (2.92)	1.73 (2.94)	0.96 (1.19)	0.0240
MYOACT scores (mean, SD)	0.22 (0.10)	0.23 (0.14)	0.21 (0.12)	0.6861
Constitutional	3.76 (2.21)	4.15 (2.49)	3.82 (2.59)	0.7663
Cutaneous	1.45 (2.04)	2.88 (2.68)	1.67 (1.71)	0.0383
Skeletal	1.31 (2.25)	0.67 (1.61)	2.04 (2.24)	0.0119
Gastrointestinal	0.67 (1.86)	0.52 (1.54)	0.44 (1.31)	0.9343
Pulmonary	3.67 (3.30)	3.27 (3.51)	4.00 (3.32)	0.6198
Cardiovascular	3.45 (2.43)	3.27 (2.91)	2.59 (2.66)	0.3602
Muscle	3.50 (2.84)	2.33 (2.92)	1.59 (2.01)	0.0135

*Notes*: Superscript letters indicate statistically significant pairwise differences.

Abbreviations: ADM, amyopathic dermatomyositis; ALP, alkaline phosphatase; ALT, alanine aminotransferase; ANA, antinuclear antibody; ARS, anti‐aminoacyl‐tRNA synthetase antibodies; ASS, anti‐synthetase syndrome; AST, aspartate aminotransferase; BMI, body mass index; CK, creatine kinase; CK‐MB, creatine kinase‐MB isoenzyme; CRP, C‐reactive protein; cTn, cardiac troponin; DM, dermatomyositis; GGT, gamma‐glutamyl transferase; HMGCR, 3‐hydroxy‐3‐methylglutaryl‐coenzyme A reductase; ILD, interstitial lung disease; IMNM, immune‐mediated necrotizing myopathy; LDH, lactate dehydrogenase; LYM, lymphocytes; MAAs, myositis‐associated autoantibodies; MB, myoglobin; MITAX, Myositis Intention to Treat Activity Index; MDA5, melanoma differentiation‐associated gene 5; MSAs, myositis‐specific autoantibodies; MYOACT, Myositis Disease Activity Assessment Visual Analogue Scales; PMN, polymorphonuclear neutrophils; RP‐ILD, rapidly progressive interstitial lung disease; SRP, signal recognition particle; TIF1γ, transcription intermediary factor 1 gamma; WBC, white blood cell count.

^a^ Cluster 1 vs. Cluster 2.

^b^ Cluster 1 vs. Cluster 3.

^c^ Cluster 2 vs. Cluster 3.

^d^ MB data are available for 93 patients.

^e^ CK‐MB data are available for 84 patients.

^f^ cTn data are available for 89 patients.

To directly link the three clusters to their corresponding clinical features and quantify these associations, we further calculated the odds ratios (ORs). Univariable logistic regression showed that patients in Cluster 1 had significantly higher odds of muscle weakness (OR = 2.61, 95% CI: 1.16–5.90, *p* = 0.021). Likewise, Cluster 2 was associated with Gottron's sign (OR = 3.81, 95% CI: 1.41–10.32, *p* = 0.009), and Cluster 3 demonstrated a notable tendency toward arthritis/arthralgia (OR = 3.82, 95% CI: 1.45–10.03, *p* = 0.007). These associations are summarized in the forest plot (Figure S1) and further support the clinical relevance of the proposed subtyping framework. Medication use did not differ significantly among the three clusters (Table S3).

### 2.3 Correlation Analysis Between IgG *N*‐Glycosylation Profiles and Clinical Features of IIMs

Building on the distinct clinical phenotypes identified by glycosylation‐driven clustering, we further used systematic correlation analyses to explore the molecular patterns underlying these associations (Figure 3B,C). Core‐fucosylated IgG2 glycoforms, particularly IgG2‐N4H5F1 and IgG2‐N5H4F1, were inversely correlated with serum CK and lactate dehydrogenase (LDH), suggesting associations with less severe muscle injury. IgG3‐N4H4F1 was also negatively associated with both the prevalence of muscle weakness and MDAAT muscle scores. In contrast, nonfucosylated IgG2‐N4H4 showed positive associations with these parameters.

Cutaneous manifestations displayed IgG subclass‐specific association patterns. IgG2‐derived glycoforms, including N3H4F1, N4H4, N4H4F1, and N4H4F1A1, were collectively associated with a lower prevalence of Gottron's sign. Conversely, higher abundances of IgG1‐N4H3F1 and IgG3‐N4H3F1 correlated with greater cutaneous involvement, suggesting subclass‐specific modulation of cutaneous inflammatory pathways. Articular involvement also showed bidirectional glycosylation associations, with IgG1‐N4H4F1 and IgG2‐N4H4 showing negative correlations with arthritis/arthralgia presence and MDAAT skeletal scores. Paradoxically, the sialylated IgG2‐N4H4F1A1 was positively correlated with joint involvement, potentially reflecting tissue‐specific activation of sialylation pathways.

Pulmonary disease activity showed glycoform‐specific associations, with IgG2‐N4H4 levels inversely correlated with MDAAT pulmonary activity scores and IgG2‐N5H4F1 positively correlated with these scores. Gastrointestinal and cardiovascular activity scores were inversely associated with IgG2‐N4H5F1, whereas IgG2‐N3H3F1 was associated with increased MDAAT cardiovascular activity scores. Nonfucosylated IgG1‐N4H4 correlated positively with biomarkers of myocardial injury, including myoglobin (MB) (*r* = 0.40), creatine kinase‐MB (CK‐MB) (*r* = 0.50), and cardiac troponin (cTn) (*r* = 0.42; all *p* < 0.001).

Laboratory biomarkers indicated a glycosylation‐mediated systemic regulation. C‐reactive protein (CRP) was positively associated with IgG2‐N3H3F1 and inversely associated with IgG2‐N4H5F1 and IgG2‐N4H5F1A1. Gamma‐glutamyl transferase (GGT) was positively correlated with core fucosylation (*r* = 0.27, *p* < 0.01), as well as with IgG2‐N4H5F1 and IgG2‐N5H4F1, whereas alkaline phosphatase (ALP) was inversely correlated with IgG2‐N4H4F1 (*r* = −0.25, *p* < 0.05), suggesting possible hepatobiliary‐related associations. Autoantibody profiles also showed mechanistically significant associations. Overall MSA positivity was inversely correlated with IgG2‐N4H4, with a particularly strong negative association for anti‐melanoma differentiation‐associated gene 5 (anti‐MDA5) antibody positivity. Anti‐MDA5 positivity was also negatively associated with IgG2‐N3H3F1 and IgG2‐N3H4F1, whereas anti‐aminoacyl‐tRNA synthetase (ARS) antibody positivity was positively associated with these two glycoforms. These differential associations suggest that IgG subclass‐specific *N*‐glycosylation patterns may differ across autoantibody‐defined immune pathways and tissue‐targeting profiles.

### 2.4 Development and Internal Validation of a Model for Predicting the Three IIM Clusters

To develop a predictive model for the three molecularly distinct IIMs subtypes associated with muscle‐dominant (Cluster 1), cutaneous‐dominant (Cluster 2), and joint‐dominant (Cluster 3) disease manifestations, we used a two‐stage feature selection approach combining clinical correlation (*p* < 0.05) and LASSO regression stability (|coefficient| > 0.5). Seven site‐specific *N‐*glycopeptides were retained as predictors: IgG3‐N4H3F1, IgG1‐N4H3F1, IgG2‐N5H5F1, IgG2‐N3H4F1, IgG2‐N3H3F1, IgG2‐N4H4, and IgG2‐N4H4F1. The multinomial logistic regression model based on these features showed strong discriminative capacity, with a likelihood ratio *χ* ^2^ of 220.79 (*p* < 0.001) and an Akaike information criterion (AIC) of 32.00, indicating good in‐sample fit while accounting for model complexity. Forest plot analyses comparing cluster‐specific associations are shown in Figure S2.

**FIGURE 4:**
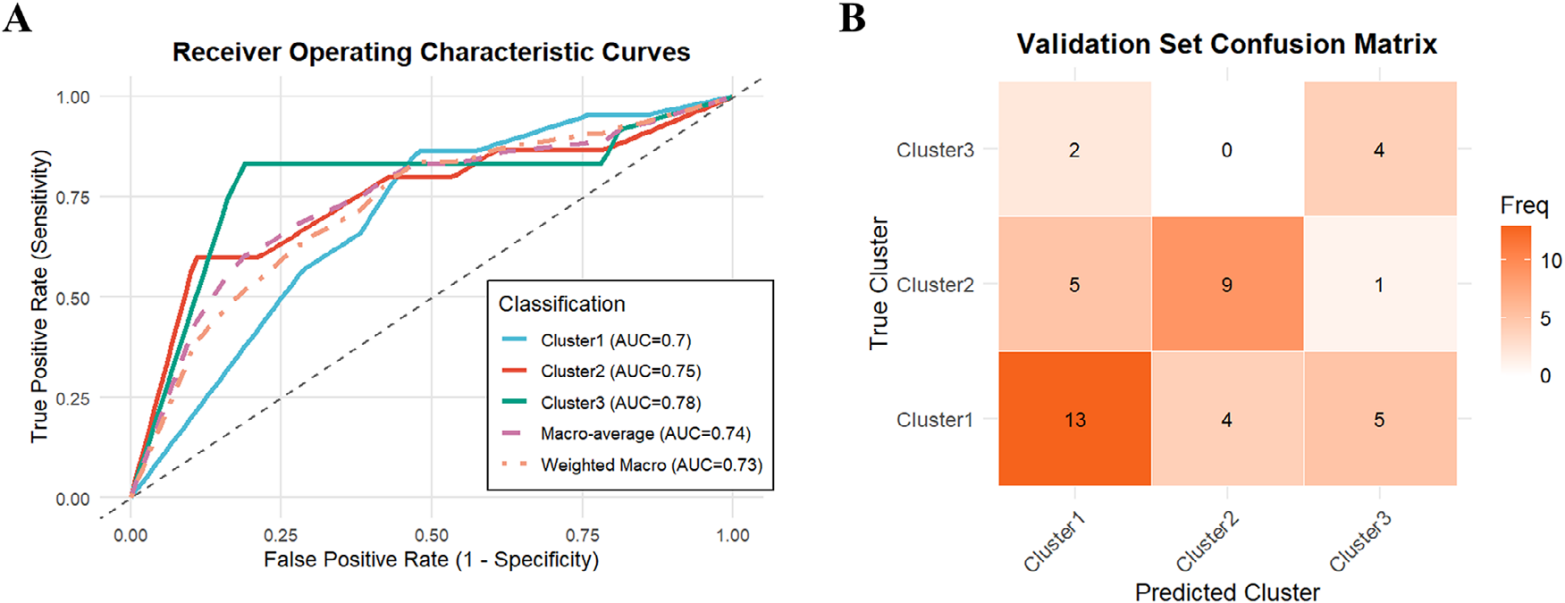
Development and internal validation of the predictive model for the three IIMs clusters based on IgG intact N‐glycopeptides. (A) Multiclass ROC analysis showing a macro‐averaged AUC of 0.74 (95% CI: 0.68–0.80), together with cluster‐specific performance highlighting differential predictive capabilities. (B) Confusion matrix heatmap showing that misclassification occurred predominantly between Clusters 1 and 3.

The multinomial logistic regression model showed consistent performance in the internal validation cohort (*n* = 43). Endotype assignment in the validation cohort was based on comparative analysis of domain‐specific MDAAT scores (Figure S3). The cluster distribution (22:15:6) did not differ significantly from that in the training set (42:33:27, *p* = 0.246), supporting the stability of the stratification approach. Multiclass receiver operating characteristic (ROC) analysis (Figure 4A) yielded cluster‐specific area under the curve (AUC) values of 0.70 (95% CI: 0.58–0.82) for Cluster 1 (muscle‐dominant), 0.75 (95% CI: 0.64–0.86) for Cluster 2 (cutaneous‐dominant), and 0.78 (95% CI: 0.67–0.89) for Cluster 3 (joint‐dominant). The macro‐averaged AUC was 0.74, and the weighted AUC was 0.73 after accounting for class imbalance, indicating similar performance despite the small number of Cluster 3 participants in the validation set (*n* = 6). Class‐specific performance metrics varied across clusters. Cluster 1 showed 59.1% sensitivity and 66.7% specificity. Cluster 2 had 60.0% sensitivity and a higher specificity of 85.7%. Cluster 3 exhibited the highest sensitivity at 66.7%, but its precision was only 40%, probably because of its small sample size. Confusion matrix analysis showed that misclassification occurred mainly between Cluster 1 and Cluster 3, suggesting partial overlap in the IgG2 glycosylation profiles associated with muscle‐ and joint‐dominant phenotypes (Figure 4B). This pattern may reflect a shared biological spectrum of musculoskeletal involvement and warrants validation in larger cohorts to determine its biological significance and refine the classifier. To further improve practical usability and directly address rapid endotype identification, we developed three endotype‐specific nomograms (Figure S4) using the same seven IgG N‐glycopeptides as raw relative‐abundance predictors. Each cluster was modeled separately in a one‐vs‐rest comparison with the other two clusters. By summing the points assigned to the individual glycopeptide abundances, users can estimate the probability of membership in the muscle‐dominant, cutaneous‐dominant, or joint‐dominant endotype.

### 2.5 Histopathological Correlations Suggest Links Among Glycosylation Profiles, Complement Deposition, and Muscle Injury in IIMs

To further assess the biological relevance of IgG *N*‐glycosylation, we evaluated complement deposition in muscle biopsy specimens using membrane attack complex (C5b‐9) immunohistochemistry (IHC). IHC results were available for 71 patients, including 50 in the training set and 21 in the validation set. Among these patients, 37 patients (52.11%) showed positive C5b‐9 deposition, indicating complement‐mediated tissue injury in a substantial proportion of the cohort.

**FIGURE 5:**
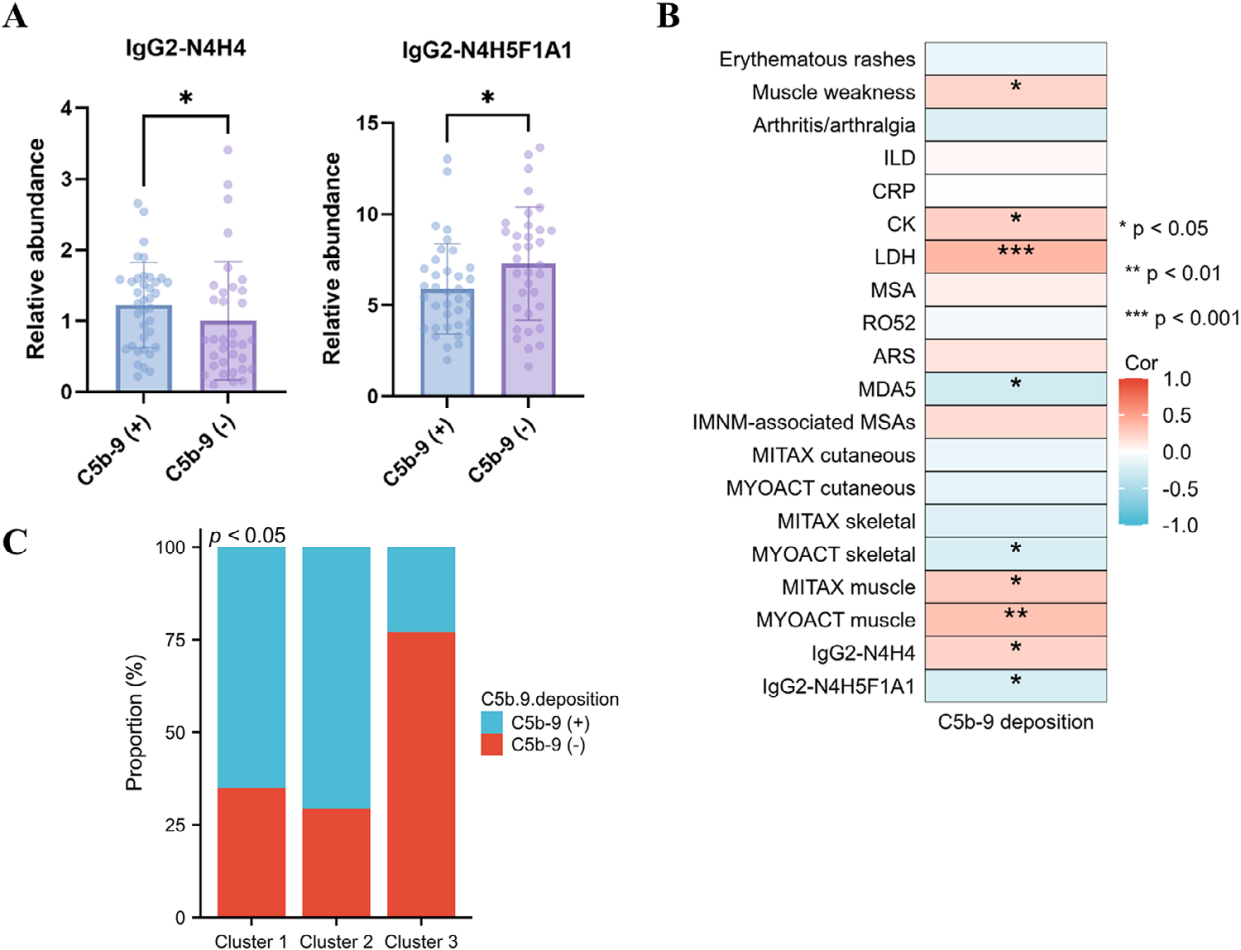
Association of IgG *N*‐glycosylation with C5b‐9 deposition and clinical features in IIMs. (A) Comparative analysis reveals differences in IgG2‐N4H4 and IgG2‐N4H5F1A1 abundances between C5b‐9(+) and C5b‐9(−) groups. (B) Correlation heatmap showing associations of C5b‐9 deposition status with clinical variables and differential IgG2 glycoform abundances. (C) Bar chart showing the proportion of C5b‐9(+) patients in each glycosylation‐defined cluster in the training cohort (*p* = 0.0249). Asterisks represent the *p*‐value as follows: *p < 0.05, **p < 0.01, ***p < 0.001, ****p < 0.0001.

Comparison of C5b‐9(+) and C5b‐9(−) patients revealed two differentially abundant IGPs (Figure 5A). Patients positive for C5b‐9 showed higher levels of IgG2‐N4H4 (1.22% ± 0.60% vs. 1.00% ± 0.83%, *p* = 0.0441), a nonfucosylated glycoform that had previously shown a positive correlation with markers of muscle injury (CK, LDH, and MDAAT‐muscle scores) in this cohort. Conversely, IgG2‐N4H5F1A1, a core‐fucosylated and sialylated glycoform that was inversely correlated with LDH and CRP, was less abundant in C5b‐9(+) patients (5.89% ± 2.47% vs. 7.28% ± 3.12%, *p* = 0.0400). Correlation analysis further supported these findings, revealing a positive association between C5b‐9 deposition status and IgG2‐N4H4 level (r = 0.241, *p* = 0.0431), and an inverse association with IgG2‐N4H5F1A1 (r = −0.242, *p* = 0.0419) (Figure 5B). These observations suggest a potential link between specific glycosylation profiles and complement deposition.

We then compared C5b‐9 positivity across the three glycosylation‐defined clusters (Figure 5C). In the training cohort, Clusters 1 and 2 exhibited significantly higher C5b‐9 positivity rates (65.00% and 70.59%, respectively) than Cluster 3 (23.08%, *p* = 0.0249). These findings were consistent with previously described cluster features, in which Cluster 1 displayed a muscle‐dominant phenotype with elevated IgG2‐N4H4, whereas Cluster 3 had a high‐sialylation profile with increased IgG2‐N4H5F1A1 and less muscle involvement. A similar pattern was observed in the validation cohort but did not reach statistical significance, likely due to limited sample size (Table S4). These cluster‐specific findings further indicate an association between IgG glycosylation profiles and tissue‐level complement deposition.

C5b‐9 positivity was also significantly correlated with several muscle‐related clinical features (Figure 5B). Positive correlations were observed with muscle weakness (*r* = 0.236, *p* = 0.0472), serum CK (*r* = 0.249, *p* = 0.0362), LDH (*r* = 0.395, *p* < 0.001), MITAX‐muscle scores (*r* = 0.283, *p* = 0.0167), and MYOACT‐muscle scores (*r* = 0.328, *p* = 0.0050). C5b‐9 positivity was negatively correlated with MYOACT‐skeletal scores (*r* = −0.235, *p* = 0.0483) and anti‐MDA5 antibody status (*r* = −0.277, *p* = 0.0193), suggesting potential differences in immunopathological features across distinct clinical subsets.

## 3 Discussion

This study provides a comprehensive characterization of IgG subclass‐specific *N*‐glycosylation in IIMs and identifies distinct *N*‐glycosylation features associated with clinical and histopathological heterogeneity. Using an integrated quantitative IGPs analysis pipeline, we identified 13 differentially abundant IGPs that distinguished patients with IIMs from healthy controls. Unsupervised clustering analysis based on these IGPs further identified three molecularly distinct subgroups: muscle‐dominant (Cluster 1), cutaneous‐dominant (Cluster 2), and joint‐dominant (Cluster 3). Each cluster had distinct clinical and serological characteristics. These findings support the potential value of IgG *N*‐glycosylation as a stratification tool that captures biologically meaningful endotypes beyond conventional clinical classification.

Overall, we observed increased core fucosylation and reduced nonfucosylated N4H4 structures in patients with IIMs, consistent with previous observations in patients with DM and RA [15, 16, 17]. These glycosylation alterations support the hypothesis that the inflammatory microenvironment may imprint specific glycosylation patterns on immunoglobulins. For instance, increased core fucosylation in our IIM cohort may be mechanistically linked to transforming growth factor‐β (TGF‐β), a cytokine implicated in myositis pathology that can transcriptionally upregulate α‐1,6‐fucosyltransferase 8 (FUT8) [18, 19]. FUT8‐mediated core fucosylation of TGF‐β receptor complexes enhances ligand binding and downstream signaling, suggesting a potential feed‐forward loop between TGF‐β signaling and core fucosylation [20, 21]. Thus, the systemic increase in fucosylated IgG may reflect the underlying profibrotic cytokine environment, although direct enzymatic regulation of this pathway in patients with IIMs remains to be demonstrated.

Subclass‐ and site‐specific glycoform analysis further revealed phenotype‐associated differences in humoral immune regulation. For the muscle‐dominant (Cluster 1) and cutaneous‐dominant (Cluster 2) clusters, the enrichment of agalactosylated (G0) and asialylated IgG glycoforms may reflect an inflammatory B‐cell differentiation program with impaired terminal galactosylation and sialylation. Previous studies have shown that B‐cell galactosyltransferase activity is reduced in RA and that inflammatory cytokines can reshape IgG glycan structures by regulating intracellular glycosyltransferases in human B cells [22, 23]. In particular, inflammatory cytokines have been shown to modulate β‐1,4‐galactosyltransferase 1 (B4GALT1) and α‐2,6‐sialyltransferase 1 (ST6GAL1) glycosylation programs during B‐cell differentiation, whereas the IL‐23/Th17 axis can suppress ST6GAL1 expression in newly differentiating antibody‐producing cells and reduce IgG Fc sialylation [23, 24].

These enzyme‐level changes provide a plausible mechanistic link between the observed IgG glycoforms and complement‐mediated tissue injury. G0 IgG exposes terminal GlcNAc residues that serve as ligands for mannose‐binding lectin (MBL) and activate the lectin complement pathway [25]. Loss of Fc sialylation may also remove an anti‐inflammatory constraint on complement activation because Fc sialylation reduces C1q binding, C3b deposition, and complement‐dependent cytotoxicity without boosting ADCC or enhancing FcγR affinity [8]. Together, these mechanisms provide a biologically plausible explanation for the prominent C5b‐9 deposition and tissue injury observed in the muscle‐dominant clusters, while recognizing that complement staining patterns vary across IIM pathological subtypes [26].

In contrast, Cluster 3 was characterized by higher sialylation, particularly enrichment of IgG2‐N4H5F1A1, and joint‐predominant symptoms. This pattern may indicate relative preservation of an ST6GAL1‐dependent sialylation program, potentially supported by B‐cell helper cytokines such as IL‐21, which can upregulate ST6GAL1 and increase IgG sialylation in human B cells [23]. Increased Fc sialylation could attenuate complement activation by limiting C1q engagement, consistent with the lower frequency of C5b‐9 deposition in this cluster [8, 27]. The enrichment of an IgG2 glycoform may also contribute to this phenotype, given that Fc‐galactosylation enhances C1q binding and complement‐dependent cytotoxicity mainly in IgG1 and IgG3, whereas galactosylation alone is insufficient to confer strong complement‐fixing activity on IgG2 [26, 28]. Consequently, the immunopathology of Cluster 3 may shift away from complement‐mediated myofiber injury toward immune‐complex‐driven articular inflammation, a mechanism well described in inflammatory arthritis [29]. The younger age of patients in Cluster 3 is also compatible with this interpretation. In the cohort‐wide correlation analysis, age was negatively associated with IgG2‐N4H5F1 and IgG2‐N4H5F1A1, but positively associated with the G0 structure (Table S5), indicating that younger patients tended to retain more highly galactosylated and sialylated IgG2 glycoforms. This finding is consistent with previous research on immunosenescence and the pro‐inflammatory environment known as “inflammaging” [30, 31].

In the internal validation cohort, the partial overlap between Cluster 1 and Cluster 3 may reflect both biological continuity across the musculoskeletal spectrum and the current limitations of predicting glycosylation‐defined endotypes. Unlike Cluster 2, both Cluster 1 and Cluster 3 are characterized by IgG2‐specific glycoform alterations, supporting partially shared IgG2‐associated humoral immune pathways involving muscle and joint inflammation. This interpretation is consistent with the clinical coexistence of myositis and arthritis frequently observed in anti‐synthetase syndrome (ASS) [32, 33]. Therefore, the seven‐glycopeptide model should be considered exploratory, and external validation in larger independent cohorts is needed to refine the boundary between muscle‐ and joint‐dominant endotypes. We also observed associations between distinct glycosylation patterns and myositis‐specific autoantibodies. IgG2‐N4H4 was inversely correlated with anti‐MDA5 antibody positivity, whereas IgG2‐N3H3F1 and IgG2‐N3H4F1 showed contrasting associations with ARS antibody status. Given that anti‐MDA5 and ARS antibodies are linked to markedly different clinical phenotypes, these findings suggest that IgG subclass‐specific *N*‐glycosylation may capture divergent MSA‐defined immune pathways [1, 34].

Histopathological evaluation further supported the clinical relevance of these glycosylation‐defined clusters. C5b‐9 deposition, a recognized marker of complement‐mediated tissue injury in DM and IMNM [35, 36], was most prevalent in the agalactosylated Clusters 1 and 2. Notably, the muscle‐injury‐associated glycoform IgG2‐N4H4 was also elevated in C5b‐9‐positive patients, whereas sialylated IgG2‑N4H5F1A1 was less abundant. Mechanistically, these observations are consistent with evidence that Fc sialylation limits complement effector activity by reducing C1q binding, downstream C3b deposition, and complement‐dependent cytotoxicity [8, 26]. These data support a direct link between circulating IgG glycosylation patterns and tissue‐level complement activation, strengthening the biological rationale for evaluating these glycoforms as integrative biomarkers.

Collectively, our study proposes a glyco‐phenotyping framework for stratifying IIMs into biologically relevant endotypes. To our knowledge, this is the first study to link IgG subclass‐specific glycosylation to data‐driven endotype discovery and histopathological findings in IIMs. Several limitations should be acknowledged. First, the retrospective cross‐sectional design and single‐time‐point sampling preclude assessment of temporal relationships or causality. Second, although internal validation provided support for our findings, the results should be interpreted cautiously given the limited sample size. Further confirmation in independent, multicenter cohorts is necessary. Third, although treatment regimens were comparable across clusters, residual confounding from prior immunosuppressive therapy could not be fully excluded because of the small number of treatment‐naïve patients and incomplete data on treatment exposure. Treatment‐naïve and longitudinal cohorts are needed to clarify treatment‐related effects. Finally, healthy controls were recruited from patients' family members, so shared environmental or genetic factors may have confounded IgG glycosylation patterns. Using unrelated, population‐based controls in future studies would better establish the specificity of disease‐associated changes.

## 4 Conclusion

In conclusion, this study provides a comprehensive characterization of IgG subclass‐specific *N*‐glycosylation in IIMs and identifies a glyco‐phenotyping framework that captures clinical and histopathological heterogeneity. Although these patterns need prospective validation, they provide a basis for future biomarker‐guided stratification and mechanistic studies in autoimmune myopathies.

## 5 Materials and Methods

### 5.1 Study Participants

This retrospective observational study comprised a cross‐sectional analysis of 168 patients diagnosed with IIMs from the Department of Rheumatology, West China Hospital, Sichuan University, between January 2022 and December 2023. The diagnostic criteria were standardized as follows: ADM was diagnosed according to the Euwer and Sontheimer's criteria [37], DM and immune‐mediated necrotizing myopathy (IMNM) were classified according to the 2017 EULAR/ACR guidelines [3], and ASS was identified using the criteria proposed by Connors et al. [38]. Two rheumatologists retrospectively reviewed and confirmed all diagnoses. During data verification, 18 patients with >10% missing laboratory data and five with concurrent malignancy were excluded. For the remaining participants, missing values were not imputed, and available‐case analysis was performed for each analysis. Variable‐specific sample sizes are reported for variables with incomplete data. HCs were recruited from family members of hospitalized patients during the same study period. Individuals were excluded if they had a current or previous diagnosis of autoimmune diseases, malignancy, or clinically significant neurological, psychiatric, or chronic systemic disorder; an active infection at enrollment; or were currently receiving immunosuppressive therapy. Individuals who were pregnant or lactating at enrollment were also excluded. HCs were selected to have an age and sex distribution comparable to that of the IIMs group. All samples were processed and analyzed using the same procedures. The final cohort included 145 patients with IIMs and 52 age‐ and sex‐comparable HCs. Ethical approval for this study was obtained from the Biomedical Research Ethics Committee of West China Hospital, Sichuan University (No. 341, 2022), and all subjects provided written informed consent.

### 5.2 Clinical Data and Sample Collection

Clinical data were systematically collected from electronic medical records at the initial hospitalization, including demographic characteristics, clinical manifestations, laboratory findings, imaging features, and muscle biopsy IHC findings. Disease activity was evaluated by two rheumatologists upon initial hospitalization using the MDAAT, from which the Myositis Intention to Treat Activity Index (MITAX), and Myositis Disease Activity Assessment Visual Analogue Scales (MYOACT) scores were calculated [39]. Venous blood samples (5 mL) were collected in ethylenediaminetetraacetic acid (EDTA) tubes, processed through centrifugation, and the resulting plasma was stored at −80°C for subsequent analysis. All clinical parameters were temporally aligned with blood sampling.

Muscle weakness was defined as a Medical Research Council (MRC) muscle strength grade of <5/5 in at least one prespecified proximal muscle group [40]. Pericardial effusion was assessed by computed tomography (CT) and/or echocardiography. Arrhythmia was diagnosed using standard 12‐lead electrocardiograms (ECGs) and/or 24‐h ambulatory ECG monitoring [41]. Autoantibody profiling included myositis‐specific autoantibodies (MSAs: anti‐ARS, anti‐MDA5, anti‐TIF1‐γ, anti‐Mi‐2, anti‐SAE‐1, anti‐NXP‐2, anti‐HMGCR, and anti‐SRP) and myositis‐associated autoantibodies (MAAs: anti‐PM/Scl, anti‐U1RNP, anti‐Ro52, anti‐SSA, anti‐SSB, and anti‐Ku). ARS antibodies were specified as anti‐Jo‐1, anti‐EJ, anti‐PL‐7, anti‐PL‐12, and anti‐OJ [42, 43].

### 5.3 Total IgG *N*‐Glycosylation Profiling

Samples were randomly renumbered before total IgG *N*‐glycosylation profiling to blind investigators to group allocation. Detailed methods for isolating, purifying, and digesting IgGs have been previously published [44]. In brief, 20 µL of plasma samples were incubated with 200 µL of binding buffer (20 mM Tris, 150 mM NaCl, pH 7.2) and 40 µL of affinity resin (Protein A/G) for 2 h at 4°C. After washing with binding buffer to remove unbound proteins, IgGs were incubated with 50 µL of 0.1 M formic acid (pH 2.5) for 5 min at 25°C on a rotator to release the captured IgGs. The eluate was then neutralized to a physiological pH. The purified IgGs were quantified through the BCA protein assay (Thermo Fisher Scientific, USA) at 562 nm.

For peptide preparation, 20 µg of purified IgGs were dissolved in 50 mM NH_4_HCO_3_ buffer (pH 8.5) and underwent denaturation (95°C, 10 min), reduction (20 mM dithiothreitol, 56°C, 30 min), and alkylation (50 mM iodoacetamide, 25°C, 30 min). The mixture was then buffer‐exchanged into 50 mM NH_4_HCO_3_ buffer (pH 8.5) using a 30 kDa ultrafiltration tube (Millipore, Bedford, MA, USA) and digested with 0.5 µg of trypsin (37°C, 1 h). The digested IgG peptides were measured using a colorimetric peptide assay (Thermo Fisher Scientific, USA) at 480 nm.

For glycoproteomic profiling, we analyzed digested peptides using an Orbitrap Fusion Lumos Tribrid Mass Spectrometer (Thermo Fisher Scientific, USA) coupled with an EASY‐nLC 1200 nanoflow high‐performance liquid chromatography (HPLC) system, as previously described [44]. Specifically, 4 µg of the peptide digest was reconstituted in 20 µL of solvent A (0.1% formic acid in water), and a 2 µL aliquot was injected onto a 20‐cm ReproSil‐Pur C18‐AQ column (1.9 µm particle size, 75 µm inner diameter; Dr. Maisch). Chromatographic separation employed a 30‐min multistep gradient at a flow rate of 350 nL/min. The mobile phase B (80% acetonitrile with 0.1% formic acid) profile was controlled as follows: 5%‐12% for the initial 2 min, increasing to 22% over 5 min, reaching 32% within the next 14 min, and finally spiking to 90% for a rapid wash.

An innovative electron‐transfer/higher energy collisional dissociation–stepped collision energy higher energy collisional dissociation (EThcD‐sceHCD) fragmentation strategy was used for data acquisition. During the electron‐transfer/higher energy collisional dissociation (EThcD) duty cycle (2 s), full mass spectrometry (MS) scans (*m*/*z* 400–1600) were acquired at a resolution of 60,000 with a custom automatic gain control (AGC) target and a maximum injection time of 50 ms. Precursor ions were isolated using a 2 *m*/*z* window and fragmented with 35% collision energy, followed by MS2 detection at 30,000 resolution (150 ms maximum injection time). The stepped collision energy HCD (sceHCD) duty cycle (1 s) employed identical MS1 settings but performed MS2 analysis at a resolution of 3000 with a 1.6 *m*/*z* isolation window. AGC targets were optimized to enhance the glycopeptide detection sensitivity. To monitor LC–MS/MS performance, an IgG mixture lysate prepared from pooled plasma of 30 patients with IIMs and 10 healthy controls was used as a quality control (QC) standard and analyzed every 20 samples. This QC procedure enabled monitoring of instrument performance and inter‐batch variation. The coefficient of variation (CV) of glycopeptide abundances across QC runs was assessed, and glycopeptides with CV <15% were considered analytically stable and retained for further analysis.

### 5.4 Data Processing and Bioinformatic Analysis

Raw data were processed using Byonic 3.10, utilizing a custom human IgG database (UniProtKB entries for IGHG1‐4), with mass tolerances of ±6 ppm for precursor ions and ±20 ppm for fragment ions. Up to two missed cleavage sites (Lysine (K) or Arginine (R)) were allowed for trypsin digestion. Fixed modifications included carbamidomethyl (C), and variable modifications included oxidation (M) and N‐terminal acetylation. A total of 182 human N‐glycans were designated as variable glycan modifications. To ensure the selected glycopeptide features were reliable and reproducible, we applied three criteria: The proteome was filtered at a 1% false discovery rate (FDR), and confident glycopeptides were required to have a Byonic score ≥ 200 and at least six amino acids. All glycopeptide spectral matches (GPSMs) were manually validated. Quantification of IGPs was performed using PANDA software (version 1.2.5) in label‐free mode. To ensure comparability across samples, the relative abundance of each glycopeptide was normalized to the total signal intensity of all IgG Fc glycopeptides detected in the same sample.

Continuous variables were presented as the mean ± standard deviation (SD) or median with interquartile range (IQR), and categorical variables as counts (%). Comparisons of continuous variables were conducted using the independent‐samples *t*‐test, one‐way analysis of variance (ANOVA), or the Kruskal–Wallis test, depending on normality and homogeneity of variance. Post hoc pairwise comparisons were adjusted for multiple testing using the Bonferroni correction following the Kruskal–Wallis test and Dunnett's test when multiple groups were compared with a single reference group in ANOVA. Categorical variables were compared using the *χ* ^2^ test or Fisher's exact test, as appropriate. Before clustering and regression analyses, relative abundances were Z‐score normalized across samples to place features on a comparable scale before multivariable analysis. To support clustering, feature selection, and model development, we randomly partitioned the IIMs cohort (*n* = 145) into a training set (*n* = 102, 70%) and a validation set (*n* = 43, 30%) using stratified sampling based on major clinical subtypes (DM, ASS, ADM, and IMNM) to ensure proportional representation. Unsupervised K‐means clustering was performed in the training set using the 13 differentially abundant IGPs between patients with IIMs and HCs, with the optimal cluster number determined by the elbow method and silhouette coefficient (> 0.2). Clustering results were visualized using PCA and heatmap plots. Pearson or Spearman's rank correlation was used to assess associations between individual glycoforms and clinical variables, as appropriate.

Univariable binary logistic regression models were used to quantify the associations between the IgG *N*‐glycosylation clusters and major clinical manifestations, expressed as ORs with 95% confidence intervals (CIs). The least absolute shrinkage and selection operator (LASSO) regression with 10‐fold cross‐validation was used to identify the glycopeptide features most informative for cluster separation. Selected glycopeptides were subsequently incorporated into a multinomial logistic regression model to predict cluster membership. Model performance was further validated in the internal validation cohort (30% of patients) using a multiclass ROC curve analysis, macro‐averaged area under the curve (AUC), and confusion matrix visualization. To facilitate rapid endotype identification, three endotype‐specific nomograms were developed as a visual scoring system. For nomogram construction, three one‐vs.‐rest logistic regression models were fitted, with each target endotype coded as 1 and the other two endotypes coded as 0. The same seven selected IgG N‐glycopeptides were entered as raw relative abundance predictors. Each nomogram estimates the probability of belonging to the corresponding endotype by summing the points assigned to individual glycopeptides.

All statistical analyses were performed, and figures were generated using IBM SPSS Statistics version 26.0, GraphPad Prism version 9.0, and R version 4.2.1.

## Author Contributions

T.W., Y. Li., Y.Z. , and Y. Liu conceived and designed the study. T.W. and Y. Li wrote the manuscript. T.W., Y.W., J.Z., L.C., and C.T. helped with the sample collection and clinical data acquisition. T.W., Y. Ling, and Yong Zhang supervised the experiments and performed the data analysis. Y. Li and C.T. contributed to project supervision and administration. Yi Zhang, Yong Zhang, and Y. Liu reviewed and edited the manuscript. Yi Zhang, Yong Zhang, and Y. Liu are the guarantors and funding recipients of this work. All authors contributed to the article and approved the submitted version.

## Funding

This work was supported by the Sichuan Science and Technology Program (2024NSFSC1762, 2026NSFSC0459), the National Natural Science Foundation of China (92478101, 82172731), the National Key R&D Program of China (2022YFF0608401), and the Special Research on Interstitial Lung Disease (2024011).

## Ethics Statement

Ethical approval was obtained from the Biomedical Research Ethics Committee of West China Hospital, Sichuan University (No. 341, 2022). Written informed consent was obtained from all participants.

## Conflicts of Interest

The authors declare no conflicts of interest.

## Supporting information




**Supporting File 1**: mco270987‐sup‐0001‐SuppMat.docx

## Data Availability

The mass spectrometry data have been deposited with the ProteomeXchange Consortium through the iProX partner repository under dataset identifier PXD058502.
