# Supplementary material for: Glycoproteomic Signatures of IgG Subclass N‐Glycosylation Reveal Molecular Endotypes in Idiopathic Inflammatory Myopathies
Source: MedComm (2020). 2026 Sep 22;7(10):e70987. doi: 10.1002/mco2.70987 (PMC13596912; doi:10.1002/mco2.70987)
Supplement: Supplementary file 1 [file MCO2-7-e70987-s001.docx]

**Supplementary Materials for**

**Glycoproteomic Signatures of IgG Subclass *N*-Glycosylation Reveal Molecular Endotypes in Idiopathic Inflammatory Myopathies**

**Short Title:**

IgG *N*-glycosylation Profiles of IIMs

**Authors:**

Tong Wu PhD^1,2, #^, Yanhong Li PhD^1,2, #^, Yingying Ling MS^1^, Yinlan Wu MS^1, 2^, Jing Zhao MS^1^, Lu Cheng MS^3^, Chunyu Tan PhD^1^, Yi Zhang PhD^4^ *, Yong Zhang PhD^1,2^ *, Yi Liu PhD^1,2,5^ *

^1^Department of Rheumatology and Immunology, Laboratory of Rheumatology and Immunology, West China Hospital, Sichuan University, Chengdu 610041, China

^2^Institute of Immunology and Inflammation, Frontiers Science Center for Disease-related Molecular Network, West China Hospital, Sichuan University, Chengdu 610041, China

^3^Department of Pulmonary and Critical Care Medicine, State Key Laboratory of Respiratory Health and Multimorbidity, West China Hospital, Sichuan University, Chengdu 610041, China

^4^Division of Pancreatic Surgery, Department of General Surgery, West China Hospital, Sichuan University, Chengdu 610041, China

^5^West China Lecheng Hospital, Sichuan University, Boao, Hainan, 571435, China

^#^These authors contributed equally.

***Corresponding authors:**

Yi Zhang (zhangyide520@wchscu.cn); Yong Zhang (zhangyong0809@wchscu.cn); Yi Liu (yiliu8999@wchscu.cn).

**Table S1.** **Comparison of the relative abundances of 11 structurally distinct IgG *N*-glycans between healthy controls and patients with IIMs**

| Glycan Composition | HC (n = 52) | IIMs (n = 145) | *P* value |
| --- | --- | --- | --- |
| N4H4, (mean, SD) | 1.26(0.38) | 0.66(0.45) | <0.0001 |
| N3H3F1, (mean, SD) | 2.93(0.42) | 3.27(1.09) | 0.0023 |
| N3H4F1, (mean, SD) | 1.24(0.24) | 1.05(0.36) | <0.0001 |
| N4H3F1, (mean, SD) | 28.66(5.81) | 34.20(8.50) | <0.0001 |
| N4H4F1, (mean, SD) | 33.13(3.51) | 32.32(4.41) | 0.2328 |
| N4H5F1, (mean, SD) | 10.88(3.39) | 8.04(3.71) | <0.0001 |
| N5H3F1, (mean, SD) | 6.38(1.46) | 6.70(2.06) | 0.2393 |
| N5H4F1, (mean, SD) | 6.16(1.60) | 5.24(1.95) | 0.0028 |
| N5H5F1, (mean, SD) | 1.46(0.54) | 0.99(0.61) | <0.0001 |
| N4H4F1A1, (mean, SD) | 2.66(1.13) | 3.21(1.01) | 0.0013 |
| N4H5F1A1, (mean, SD) | 5.26(2.11) | 4.33(2.19) | 0.0022 |

Note: N: N-acetylhexosamine; H: hexose; F: fucose; A: N-acetylneuraminic acid.

**Table S2.** **Relative abundances of IgG glycosylation features across IIMs subgroups**

| Composition  (mean, SD) | ADM (n = 24) | ASS (n = 35) | DM (n = 62) | IMNM (n = 24) | *P* value |
| --- | --- | --- | --- | --- | --- |
| Core fucosylation | 99.55(0.35) | 99.30(0.39) | 99.47(0.38) | 98.87(0.49) | <0.0001 |
| Sialylation | 6.90(2.43) | 6.43(2.77) | 8.18(3.03) | 8.15(2.96) | 0.0156 |
| IgG1-N4H3F1 | 4.28(3.55) | 5.16(3.95) | 3.73(2.75) | 2.67(2.01) | 0.0269 |
| IgG1-N4H4F1 | 4.45(3.37) | 4.64(3.38) | 4.60(3.24) | 3.96(2.91) | 0.8511 |
| IgG2-N3H3F1 | 3.15(0.97) | 4.19(1.08) | 2.86(0.98) | 3.08(0.69) | <0.0001 |
| IgG2-N3H4F1 | 0.94(0.36) | 1.15(0.31) | 1.00(0.38) | 1.14(0.34) | 0.0542 |
| IgG2-N4H3F1 | 26.86(4.75) | 30.03(7.16) | 25.19(6.18) | 25.94(6.99) | 0.0051 |
| IgG2-N4H4 | 0.45(0.35) | 0.70(0.39) | 0.53(0.38) | 1.13(0.49) | <0.0001 |
| IgG2-N4H4F1 | 22.89(4.18) | 21.22(4.91) | 24.27(4.12) | 28.27(4.35) | <0.0001 |
| IgG2-N4H4F1A1 | 3.08(0.96) | 2.83(0.95) | 3.39(1.05) | 3.45(0.94) | 0.0321 |
| IgG2-N4H5F1 | 7.59(3.24) | 6.78(3.45) | 8.73(4.02) | 8.55(3.30) | 0.0702 |
| IgG2-N4H5F1A1 | 3.81(1.74) | 3.60(2.06) | 4.80(2.24) | 4.70(2.33) | 0.0314 |
| IgG2-N5H3F1 | 7.62(2.39) | 6.76(1.71) | 6.80(1.93) | 5.40(1.96) | 0.0017 |
| IgG2-N5H4F1 | 5.54(1.96) | 4.63(1.93) | 6.65(1.96) | 4.80(1.74) | 0.0464 |
| IgG2-N5H5F1 | 0.87(0.52) | 0.79(0.62) | 1.13(0.63) | 1.04(0.57) | 0.0444 |
| IgG3-N4H3F1 | 3.71(2.48) | 4.06(3.48) | 3.33(2.80) | 2.58(1.63) | 0.1900 |
| IgG3-N4H4F1 | 4.74(3.31) | 3.45(2.84) | 3.99(2.89) | 3.29(2.91) | 0.3001 |

Note: ADM: amyopathic dermatomyositis; ASS: anti-synthetase syndrome; DM: dermatomyositis; IMNM: immune-mediated necrotizing myopathy. N: N-acetylhexosamine; H: hexose; F: fucose; A: N-acetylneuraminic acid.

| Variables | Cluster 1 (n = 42) | Cluster 2 (n = 33) | Cluster 3 (n = 27) | *P* value |
| --- | --- | --- | --- | --- |
| Treatment naïve, n (%) | 16 (38.10) | 13 (39.39) | 7 (25.93) | 0.5278 |
| Treatment during this admission |  |  |  |  |
| GC <50mg/day prednisone equivalent, n (%) | 20 (47.62) | 15 (45.45) | 14 (51.85) | 0.9077 |
| GC ≥50mg/day prednisone equivalent, n (%) | 5 (11.90) | 3 (9.09) | 5 (18.52) | 0.5667 |
| HCQ, n (%) | 6 (14.29) | 3 (9.09) | 4 (14.81) | 0.8044 |
| CTX, n (%) | 9 (21.43) | 7 (21.21) | 7 (25.93) | 0.9112 |
| CNI, n (%) | 11 (26.19) | 4 (12.12) | 6 (22.22) | 0.3199 |
| Other csDMARDs¶, n (%) | 7 (16.67) | 3 (9.09) | 2 (7.41) | 0.5771 |
| JAK inhibitors¶¶, n (%) | 6 (14.29) | 2 (6.06) | 2 (7.41) | 0.5377 |
| IVIg, n (%) | - | - | 2 (7.41) | 0.2481 |
| Anti-pulmonary fibrosis drugs¶¶¶, n (%) | 8 (19.05) | 4 (12.12) | 5 (18.52) | 0.7504 |

**Table S3. The medication regimens across the three IIMs clusters**

Note: ¶: Other csDMARDs included methotrexate and mycophenolate mofetil.

¶¶: JAK inhibitors included baricitinib and tofacitinib.

¶¶¶: Anti-pulmonary fibrosis drugs included nintedanib and pirfenidone.

GC, glucocorticoids; HCQ, hydroxychloroquine; CTX, cyclophosphamide; CNI, calcineurin inhibitor; csDMARDs, conventional synthetic disease-modifying anti-rheumatic drugs; IVIg, intravenous immunoglobulin.

**Table S4. Distribution of C5b-9-positive cases across glycosylation-defined clusters in the validation cohort (n = 21)**

| Cluster | Total patients (n) | C5b-9 positive (n) | C5b-9 positive (%) | *P* value |
| --- | --- | --- | --- | --- |
| Cluster 1 | 13 | 7 | 53.85% |  |
| Cluster 2 | 5 | 1 | 20.00% |  |
| Cluster 3 | 3 | 1 | 33.33% |  |
| Total | 21 | 9 | 42.86% | 0.5621 |

Note: *p*-value derived from Fisher’s exact test.

**Table S5. Correlations between age and the relative abundances of intact IgG *N*-glycopeptides in the training set (n = 102)**

| Glycopeptide | Correlation coefficient | *p* value |
| --- | --- | --- |
| IgG2-N3H3F1 | 0.29 | 0.0035 |
| IgG2-N3H4F1 | –0.04 | 0.7204 |
| IgG1-N4H3F1 | 0.26 | 0.0073 |
| IgG3-N4H3F1 | 0.20 | 0.0442 |
| IgG2-N4H4 | 0.15 | 0.1429 |
| IgG1-N4H4F1 | 0.17 | 0.0795 |
| IgG2-N4H4F1 | 0.09 | 0.3767 |
| IgG3-N4H4F1 | 0.13 | 0.2064 |
| IgG2-N4H4F1A1 | –0.05 | 0.6115 |
| IgG2-N4H5F1 | –0.48 | <0.0001 |
| IgG2-N4H5F1A1 | –0.37 | 0.0001 |
| IgG2-N5H4F1 | –0.29 | 0.0033 |
| IgG2-N5H5F1 | –0.49 | <0.0001 |

Note: Correlation coefficients were calculated using Pearson's method. Statistical significance was defined as *P* < 0.05. N: N-acetylhexosamine; H: hexose; F: fucose; A: N-acetylneuraminic acid.

**Figure S1. Risk associations between glycosylation-defined clusters and representative clinical phenotypes**

Note: Forest plot showing the associations between glycosylation-defined clusters and representative clinical phenotypes. Cluster 1 was associated with muscle weakness (OR = 2.61, 95% CI: 1.16–5.90, *p* = 0.021), Cluster 2 with Gottron’s sign (OR = 3.81, 95% CI: 1.41–10.32, *p* = 0.009), and Cluster 3 with arthritis/arthralgia (OR = 3.82, 95% CI: 1.45–10.03, *p* = 0.007). OR, odds ratio; CI, confidence interval.

**Figure S2. Multinomial logistic regression coefficients for glycopeptide-defined endotype classification**

Note: Coefficient plot showing the contribution of seven selected IgG N-glycopeptide features to the multinomial logistic regression model for classifying candidate glycosylation-defined subgroups. Cluster 1 was used as the reference category, and coefficients are shown for Cluster 2 and Cluster 3. Points represent regression coefficients, and horizontal error bars indicate 95% confidence intervals. Positive coefficients indicate increased likelihood of assignment to the corresponding cluster relative to Cluster 1, whereas negative coefficients indicate decreased likelihood.

**Figure S3. The cluster assignment process for validation cohort patients (n=43)**

Note: Clinical reference labels were assigned according to the highest MDAAT domain score: muscle (Cluster 1; n = 22), cutaneous (Cluster 2; n = 15), or joint (Cluster 3; n = 6). These clinically anchored labels were used for split-sample evaluation of the glycopeptide classifier.

**Figure S4. The visual scoring system for the three IIMs endotypes**

Note: Endotype-specific nomograms were developed under a one-vs-rest logistic regression framework for visual estimation of the probability of belonging to Cluster 1 (muscle-dominant) (A), Cluster 2 (cutaneous-dominant) (B), and Cluster 3 (joint-dominant) (C). The nomograms were generated using the same seven IgG N-glycopeptides selected for the primary prediction model.
